# Assessment of Interleukins IL-4, IL-6, IL-8, IL-10 in Acute Urticaria

**DOI:** 10.14740/jocmr1645w

**Published:** 2014-02-06

**Authors:** Jordan Papadopoulos, Anthony Karpouzis, John Tentes, Constantin Kouskoukis

**Affiliations:** a Department of Dermatology, Faculty of Medicine, Democritus University of Thrace, Greece; b Laboratory of Biochemistry, Faculty of Medicine, Democritus University of Thrace, Greece

**Keywords:** Acute urticaria pathogenesis, Interleukins, IL-4, IL-6, IL-8, IL-10

## Abstract

**Background:**

Urticaria is a skin disease that affects approximately 5% of the general population and manifests itself, not only as an acute but also as a chronic disease. The etiology of the disease varies as well as its clinical manifestations which extend from the presence of urticarial hives to the potentially mortal angioedema. There is a great interest to the disease not only due to its special clinical manifestation but also due to its pathogenetic mechanism. New data in the medical bibliography support the participation of interleukins (ILs) in the pathophysiology of urticaria. The aim of the study is to contribute in the comprehension of possible participation of certain ILs in the pathogenesis of acute urticaria.

**Methods:**

Our study concerns four ILs, IL-4, IL-6, IL-8 and IL-10, simultaneously and their quantitative changes during the acute phase of urticaria as well as 2 weeks after drug administration. Moreover, ILs levels of patients were compared with those of matched healthy controls. All measurements have been done by the ELISA method. The statistical analysis was done by SPSS.

**Results:**

The results present increased levels (in 51 patients vs. 22 matched healthy controls) of all four ILs during the acute phase. Especially for IL-4 this increase was statistically very significant (P < 0.001). Statistically marginally significant decrease was also observed for IL-10 concentrations (P < 0.059), for the two blood samples (acute phase and 2 weeks later).

**Conclusion:**

It is suggested by the present study that certain ILs might play an important role in the pathogenetic mechanism of urticaria. IL-4 and IL-10 participation seems to be relatively more significant. Possibly, ILs, liberated by mast cells, induce an influx of leukocytes in the dermis, therefore participating in the development of acute urticaria inflammation.

## Introduction

Urticaria is a skin disease characterized by the appearance of itchy wheals. These lesions are caused by localized edema. Angioedema is the more severe form of urticaria. The disease is distinguished in acute, relapsing (episodes of acute urticaria with intervals between them) and chronic (episodes of acute eurticaria without intervals) form. There are many etiologic factors such as foods, drugs, infections and inhalants. The major mediator for the urticarial reactions is histamine and possibly leukotrienes, proteases, kinines, TNF-α and GM-CSF [1, 2].

Throughout the body encountered mast cell degranulation may occur: 1) when an allergen binds on the mast cell site of interaction between Ce3 IgE domain and IgE receptor; 2) by anti-FceRI antibodies; 3) by cross-linking of adjacent FceRI; 4) by allergen-induced binding between receptor and relevant bound IgE; 5) non-immunologic stimuli (C5a anaphylotoxin, opioids, stem cell factor as well as neuropeptides as substance, P via direct stimulation). Mast cell preformed cytokines, interleukins (ILs), leukotrienes and TNF-α are responsible for the migration of inflammatory cells from the blood into the urticarial lesion [1-4].

New data in the medical bibliography support the participation of ILs in the pathophysiology of urticaria [3, 4]. The present study refers to four ILs: IL-4, IL-6, IL-8 and IL-10.

IL-4 has been measured in many studies involving urticaria [5-8].

It was originally named B-cell growth factor. It is released in the skin by keratinocytes or mast cells. It acts mainly on immunocompetent cells, and induces proliferation of activated mature T cells. It is also important in the differentiation towards a Th2 T-cell subtype. IL-4 stimulates mast cell growth and regulates IgE synthesis.

IL-6 is mainly produced by monocytes, bone marrow cells, fibroblasts, endothelial cells, some T cells, B cells and keratinocytes. It is involved in the regulation of the function of T, B and NK cells. It also plays an essential role in antibody production by B cells. IL-6 mediates activation, growth and differentiation of T cells. Thus, IL-6 is an “early” pro-inflammatory cytokine. IL-6 serum levels have also been studied in patients with urticaria [9-12].

IL-8 is the most potent chemokine for attracting neutrophils and has been studied in several cases of urticaria [13, 14]. IL-8 (produced by mast cells) upregulates (in synergy with TNF-α) the expression of adhesion molecules on endothelial cells and encourages migration of circulating inflammatory cells from blood into urticarial lesions [4].

IL-10 (produced by both subsets of Th cells, activated monocytes, and in HIV seropositive subjects, by B cells as well) is an important cytokine for the regulation of antigen presentation and suppression of Th1 and Th2 cytokine production [15-17]. IL-10 expression in urticarial lesions is characterized by a wide range (ascertained by PCR) [18].

## Methods

### Patients and controls

The study was carried out from October 2005 to September 2008 and involves all patients with urticaria that presented in the outpatient clinic of the Dermatology Department of the Faculty of Medicine of the Democritus University of Thrace in the corresponding University Hospital.

Work procedures were consistent with Helsinki Declaration Principles. There is no conflict of interest, regarding any of the authors.

Patients, who presented with urticaria, were examined and asked thoroughly about the onset and type of symptoms and predisposing factors of the specific episode. Antihistamine treatment was stopped at least 4 days before blood-taking. These patients were administered medication and asked to come for a follow-up examination after 2 weeks. During this second visit to our clinic, a second blood sample was taken from the patients.

The study involves 51 patients (40 women and 11 men) with mean age of 42.27 years, range 10 - 82 years old. All our acute urticaria cases were of grade 2 (according to Sabroe et al’s classification) [19], at the time when blood samples were collected. In our group, there were no cases associated either with angioedema or with such a gravity that might impose the hospitalization of patients. All of our patients (by taking in consideration that did not take drugs influencing blood coagulation) responded efficiently to treatment including once only intramuscular injection of acetic betamethasone + phosphoric betamethasone and POs administration of ketotifen (1 tablet of 1 mg × 2 × 7 days, 1 tablet per night for 7 days more). Control blood samples were also obtained from 22 healthy volunteers (15 women and 7 men; mean age: 57.77 years, range 23-89). Only 23 patients came for a follow-up blood test.

We have excluded patients who had already taken medication before their visit in our clinic.

Data collection and analysis have been done according to the rules of the good clinical practice.

### Measurement of ILs levels

Serum IL-4, IL-6, IL-8 and IL-10 levels were measured by enzyme-linked immunosorbent assay (ELISA) in pg/mL.

The name of ELISA kit, company name and kit sensibility are as follows: R&D Systems, Inc., Minneapolis, MN, USA. 1) Human IL-4 Quantikine ELISA kit (catalogue number: D4050). Intra-assay precision: 3.4%. Inter-assay: 7.8%. Sensitivity: 10 pg/mL. Range 31.2 - 2,000 pg/mL. 2) Human IL-6 Quantikine ELISA kit (catalogue number: D6050). Coefficient of variation: Intra-assay precision: 1.6%. Inter-assay precision: 3.3%. Sensitivity: 0.7 pg/mL. Range: 3.12 - 300 pg/mL. 3) Human CXCL 8/IL-8 Quantikine ELISA kit (catalogue number: D8000 C). Intra-assay: 5.4%. Inter-assay: 7.40%. Sensitivity: 7.5 pg/mL. Range: 31.2 - 2,000 pg/mL. 4) Human IL-10 Quantikine HS ELISA kit (catalogue number: HS100 C). Intra-assay: 5.8%. Inter-assay: 7.8%. Sensitivity: 0.17 pg/mL. Range: 0.78 - 50 pg/mL.

The first blood sample was taken in the acute phase. After 2 weeks, we proceeded in a second blood-taking from the patients. In the meanwhile, the patients took medication for the urticarial episode. The blood was taken from peripheral vein into heparinized vacutainer tubes. All blood samples were centrifuged in 2,500*g* for 10 min and the serum was stored in -80 °C. For each patient we took two tubes with serum.

ELISAs were performed in flat bottom 96-well plates which permit high throughput results. The bottom of each well was coated with a protein. The serum was incubated in a well. After a few time (1 h) in room temperature, the serum was removed and the samples were washed off with a series of buffer rinses. Then we put enzymes (peroxidase) for 30 min to metabolize colorless substrates into colored products. The final step was the addition of the enzyme substrate and the production of colored product in wells.

When the enzyme reaction was complete, the entire plate was placed into a plate reader and the optical density is determined for each well at 405 nm.

The statistic analysis of all the data was accomplished with SPSS 15 using non-parametric Wilcoxon signed rank tests. Our aim was to evaluate if there is a significant increase in IL levels in the acute phase of urticaria, if there is a significant difference between the first and the second blood-taking, as well as if the presence of positive history of urticaria affects significantly the levels of ILs.

## Results

The serum levels of the ILs in the first blood sample of the patients during the acute phase are IL-4: 0.632 pg/mL, IL-6: 1.689 pg/mL, IL-8: 1,478 pg/mL and IL-10: 0.967 pg/mL.

**Figure 1:**
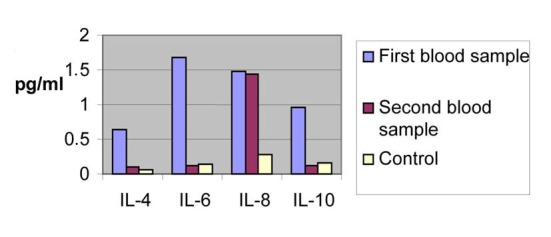
Concentrations from the first and the second blood samples of the patients compared to the measurements of the healthy volunteers.

The serum levels of the ILs of the healthy volunteers are IL-4: 0.065 pg/mL, IL-6: 0.146 pg/mL, IL-8: 0.273 pg/mL and IL-10: 0.151 pg/mL (Fig. 1).

We observe that the serum levels of the all ILs of the patients are higher in the acute phase compared to the serum levels of the volunteers.

**Figure 2:**
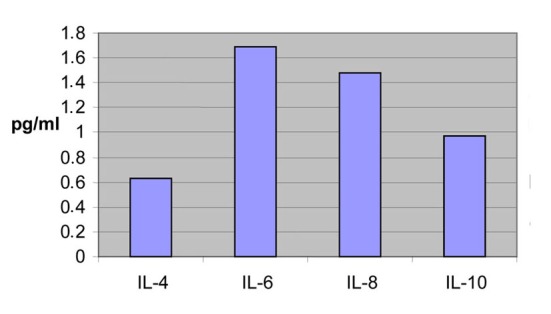
Concentrations from the first blood sample of the patients.

Especially for IL-4 this increase was statistically very significant (P = 0.001) (Fig. 2).

We also observed that the serum level of IL-10 of the patients between the first blood-taking (acute phase) and the second blood-taking (2 weeks later) was decreased. This decrease is statistically significant (P = 0.059).

**Figure 3:**
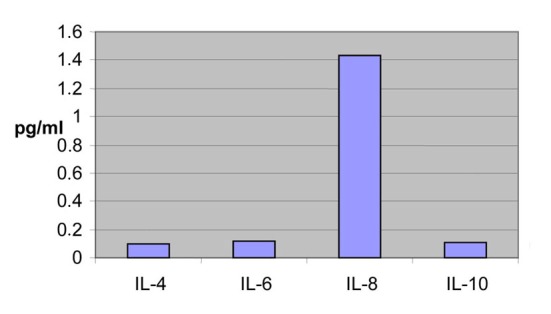
Concentrations from the second blood sample of the patients.

**Figure 4:**
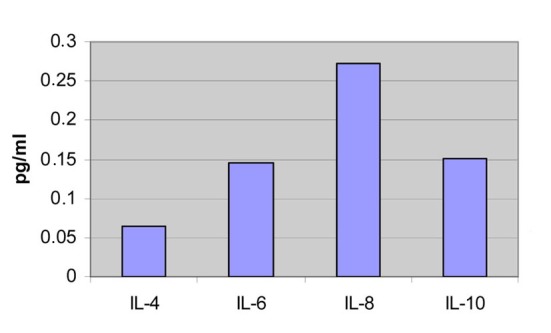
Measurements of the healthy volunteers.

The other ILs range almost in the same levels between the two blood-takings (Fig. 3, 4).

## Discussion

The exact mechanisms leading to urticaria are not known in detail and the involvement of ILs in the pathogenesis of urticaria is subject for discussion. Various studies have been recently performed to identify those mechanisms. ILs are potent anti-inflammatory cytokines which are produced by a variety of cells, specifically T_H_2 cells, and by macrophages as well as by mast cells in the periphery. Since now there is no study in the literature describing the levels of all four ILs (IL-4, IL-6, IL-8 and IL-10) in the patients with urticaria. There have been studies evaluating the levels of isolated ILs in patients with urticaria, but none of them compared the levels of the four ILs in the same patient (IL-4, IL-6, IL-8 and IL-10) [5-13, 15-18].

Moreover, in our study the serum levels of all four ILs were compared not only to the serum levels of the ILs in the control group, but also to the serum levels of the same patient at two different points of time. The first blood sample was taken at the acute phase during the patient’s first visit at the hospital and the second one was taken 2 weeks later.

If we compare the IL levels of the patients and the control group, we observe that concentration of all ILs was elevated in patients at the acute phase in a different degree between the four molecules.

This increase was statistically very significant (P < 0.001) for IL-4 in particular.

This result might indicate that IL-4 is more important during the acute phase of urticaria, among the four ILs.

The decrease of IL-10 levels (P < 0.059) at the acute phase in comparison with 2 weeks later, perhaps is more specific compared to the other ILs.

In our study, there were no cases associated either with angioedema or with such a gravity that might impose the hospitalization of patient. Our data permitted no correlation of case gravity with any IL serum level [20]. All of the patients responded satisfactorily to treatment with once only intramuscular injection of acetic betamethasone + phosphoric betamethasone and POs administration of ketotifen (1 tablet of 1 mg × 2 for 7 days, 1 tablet per night for 7 days more).

### Conclusion

This study suggested that at least IL-4 and IL-10 constitute inflammation mediators in acute urticaria and their levels in the serum possibly have a useful prognostic interest.

## References

[1] Zuberbier T, Maurer M (2007). Urticaria: current opinions about etiology, diagnosis and therapy. Acta Derm Venereol.

[2] Ghosh S (2009). What'S new in urticaria?. Indian J Dermatol.

[3] Hennino A, Berard F, Guillot I, Saad N, Rozieres A, Nicolas JF (2006). Pathophysiology of urticaria. Clin Rev Allergy Immunol.

[4] Poonawalla T, Kelly B (2009). Urticaria : a review. Am J Clin Dermatol.

[5] Mohamed RW, Fathy A, el-Sayed AE (2003). Increased circulating FcepsilonRII-bearing B-lymphocytes and serum levels of IL-4 in non-autoreactive chronic idiopathic urticaria. Egypt J Immunol.

[6] Confino-Cohen R, Goldberg A, Aharoni D, Naiman L, Buchs A, Weiss M, Weissgarten J (2004). Low stimulated IL-4 secretion in PBMC from patients with chronic idiopathic urticaria. Cytokine.

[7] Ferrer M, Luquin E, Sanchez-Ibarrola A, Moreno C, Sanz ML, Kaplan AP (2002). Secretion of cytokines, histamine and leukotrienes in chronic urticaria. Int Arch Allergy Immunol.

[8] Asero R, Tedeschi A, Lorini M, Salimbeni R, Zanoletti T, Miadonna A (2001). Chronic urticaria: novel clinical and serological aspects. Clin Exp Allergy.

[9] Fujii K, Ohgou N (2004). Up-regulation of IL-6 mRNA in peripheral blood mononuclear cells of patients with acute urticaria. J Dermatol.

[10] Tillie-Leblond I, Gosset P, Janin A, Salez F, Prin L, Tonnel AB (1998). Increased interleukin-6 production during the acute phase of the syndrome of episodic angioedema and hypereosinophilia. Clin Exp Allergy.

[11] Lin RY, Trivino MR, Curry A, Pesola GR, Knight RJ, Lee HS, Bakalchuk L (2001). Interleukin 6 and C-reactive protein levels in patients with acute allergic reactions: an emergency department-based study. Ann Allergy Asthma Immunol.

[12] Kasperska-Zajac A, Brzoza Z, Rogala B (2007). Plasma concentration of interleukin 6 (IL-6), and its relationship with circulating concentration of dehydroepiandrosterone sulfate (DHEA-S) in patients with chronic idiopathic urticaria. Cytokine.

[13] Choi SJ, Ye YM, Hur GY, Shin SY, Han JH, Park HS (2008). Neutrophil activation in patients with ASA-induced urticaria. J Clin Immunol.

[14] Santos JC, de Brito CA, Futata EA, Azor MH, Orii NM, Maruta CW, Rivitti EA (2012). Up-regulation of chemokine C-C ligand 2 (CCL2) and C-X-C chemokine 8 (CXCL8) expression by monocytes in chronic idiopathic urticaria. Clin Exp Immunol.

[15] Irinyi B, Aleksza M, Antal-Szalmas P, Sipka S, Hunyadi J, Szegedi A (2002). Cytokine production of CD4+ and CD8+ peripheral T lymphocytes in patients with chronic idiopathic urticaria. Acta Derm Venereol.

[16] Chodorowska G, Czelej D, Krasowska D, Pietrzak A (2003). Plasma activity of interleukin-10 in drug-induced cutaneous reactions. Ann Univ Mariae Curie Sklodowska Med.

[17] Czelej D, Chodorowska G, Lechowska-Mazur I (2003). Drug-induced urticaria--activity of selected cytokines and acute phase proteins in plasma. Ann Univ Mariae Curie Sklodowska Med.

[18] Garcia E, Duarte S, Calderon C, Gonzalez JM, Cuellar A, Gomez A, Halpert E (2011). [Expression of IL-10, IL-4 and IFN-gamma in active skin lesions of children with papular urticaria]. Biomedica.

[19] Sabroe RA, Fiebiger E, Francis DM, Maurer D, Seed PT, Grattan CE, Black AK (2002). Classification of anti-FcepsilonRI and anti-IgE autoantibodies in chronic idiopathic urticaria and correlation with disease severity. J Allergy Clin Immunol.

[20] Fujii K, Konishi K, Kanno Y, Ohgou N (2001). Acute urticaria with elevated circulating interleukin-6 is resistant to anti-histamine treatment. J Dermatol.

